# Tri-Lineage Differentiation Potential of Osteosarcoma Cell Lines and Human Bone Marrow Stromal Cells from Different Anatomical Locations

**DOI:** 10.3390/ijms24043667

**Published:** 2023-02-11

**Authors:** Hannah L. Smith, Juliet C. Gray, Stephen A. Beers, Janos M. Kanczler

**Affiliations:** 1 Antibody and Vaccine Group, Centre for Cancer Immunology, Cancer Sciences Unit, Faculty of Medicine, University of Southampton, Southampton General Hospital, Southampton SO16 6YD, UK; 2 Bone and Joint Research Group, Institute of Developmental Sciences, Human Development and Health, Faulty of Medicine, University of Southampton, Southampton General Hospital, Southampton SO16 6YD, UK

**Keywords:** osteosarcoma, bone marrow, tri-lineage differentiation, human bone marrow stromal cells

## Abstract

The bone cancer osteosarcoma, found mainly in adolescents, routinely forms around the growth plate/metaphysis of long bones. Bone marrow composition changes with age, shifting from a more hematopoietic to an adipocyte-rich tissue. This conversion occurs in the metaphysis during adolescence, implicating a link between bone marrow conversion and osteosarcoma initiation. To assess this, the tri-lineage differentiation potential of human bone marrow stromal cells (HBMSCs) isolated from the femoral diaphysis/metaphysis (FD) and epiphysis (FE) was characterized and compared to two osteosarcoma cell lines, Saos-2 and MG63. Compared to FE-cells, FD-cells showed an increase in tri-lineage differentiation. Additionally, differences were found between the Saos-2 cells exhibiting higher levels of osteogenic differentiation, lower adipogenic differentiation, and a more developed chondrogenic phenotype than MG63, with the Saos-2 being more comparable to FD-derived HBMSCs. The differences found between the FD and FE derived cells are consistent with the FD region containing more hematopoietic tissue compared to the FE. This may be related to the similarities between FD-derived cells and Saos-2 cells during osteogenic and chondrogenic differentiation. These studies reveal distinct differences in the tri-lineage differentiations of ‘hematopoietic’ and ‘adipocyte rich’ bone marrow, which correlate with specific characteristics of the two osteosarcoma cell lines.

## 1. Introduction

Osteosarcoma is a rare form of bone cancer with a peak of incidence found during adolescence, roughly 10 to 20 years of age [1]. Development of this tumor occurs most notably in the metaphysis of the long bones including the proximal humerus, distal femur, and proximal tibia [2,3,4]. The exact mechanism of osteosarcoma development is still poorly understood, although there is strong evidence suggesting that these tumors result from an unidentified mutation in osteoblast precursor cells [5], which may occur during conversion of the bone marrow.

Bone marrow is the main component in the medullary cavity of bone, which include stem cell-like precursors known as mesenchymal stem cells (MSCs). Although MSCs are not accurately defined they are known for their ability to differentiate into structural bone, osteoblasts, adipocytes and chondrocytes [6], playing a fundamental role in bone growth, regeneration, and repair. Bone marrow is routinely separated into two categories, namely, ‘red’ and ‘yellow’, with the coloration resulting from their differing cellular composition. Nuclear magnetic resonance (NMR) imaging techniques have demonstrated that red bone marrow is composed of approximately 60% hematopoietic tissue and 40% adipose tissue [7,8]. Hematopoietic tissue contains a heterogenous population that is able to differentiate into red blood cells and platelets, as well as white blood cells (both myeloid and lymphoid) required for an effective immune system. Hematopoiesis occurs within a stromal cell framework that includes endothelial and mesenchymal cells, both of which play a role in hematopoiesis regulation [9,10].

**Figure 1:**
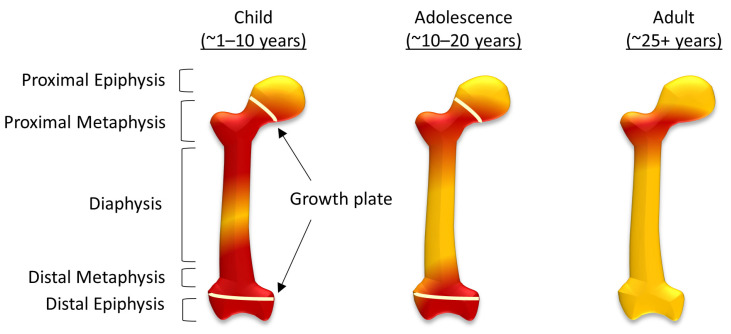
The conversion of red to yellow bone marrow in the femur. The distribution of red and yellow bone marrow in children, adolescents and in adulthood, showing structural components of the femur including the diaphysis, metaphysis, and epiphysis (both sulphur proximal and distal) and the growth plates during childhood and adolescence.

In comparison, ‘yellow’ bone marrow is composed of around 95% adipocytes [7,8], and although their function is still not completely understood, there have been data to suggest adipocytes can also regulate hematopoiesis, as well as a metabolism [11,12]. During human fetal development ‘red’ bone marrow occupies all bones, and as the body develops this gradually converts to ‘yellow’ bone marrow [7,8]. In the femur, this process starts in the diaphysis and progresses both proximally and distally within the bone (Figure 1). By adulthood, ‘red’ marrow is only found in the proximal metaphysis of the femur [8], although an increase in demand for hematopoietic cells can result in the reconversion of ‘yellow’ bone marrow to ‘red’. This can be caused by severe blood loss [13], chronic anemia, as well as non-medical conditions associated with stress, including smoking and, in some cases, it is associated with athletes [14]. Osteosarcoma typically forms around the growth plates, which, during adolescence, are also the areas of ‘red’ to ‘yellow’ bone marrow conversion [7].

To consider the relationship between osteosarcoma initiation and bone marrow conversion, differences between the ‘red’ and ‘yellow’ bone marrow were characterized in vitro. Primary human bone marrow stromal cells (HBMSCs) were isolated from the femoral epiphysis (FE) and femoral metaphysis/diaphysis (FD) of patients undergoing hip replacement surgery. These cells underwent tri-lineage differentiation, resulting in distinct characteristics for the two subsets of bone marrow, which included increases in osteogenic and chondrogenic potential in the FD cells compared to the FE. Two osteosarcoma cell lines were also assessed for tri-lineage differentiation and compared against the HBMSCs. These data showed that the osteosarcoma cell lines had differing phenotypes from each other, which partially recapitulated characteristics shown by the FD and FE HBMSCs. This supports a possible link between the stage of bone marrow conversion and the development of osteosarcoma.

## 2. Results

### 2.1. Osteogenic Differentiation of HBMSCs

**Figure 2:**
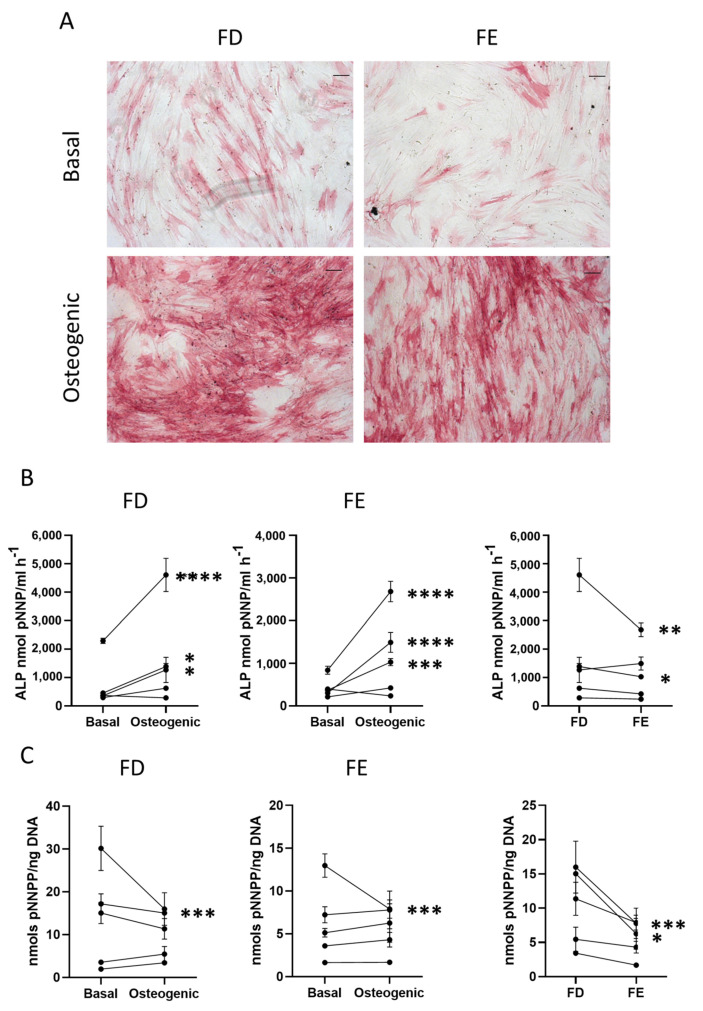
Early osteogenic differentiation of HBMSCs from two regions in the femur. (**A**) Representative images of ALP staining (pink/red) of HBMSCs from two femur regions, FD and FE. Cells were cultured in basal or osteogenic I media for seven days. Scale Bar= 100 µm. (**B**) ALP analysis of HBMSCs, FD and FE. (**C**) ALP specific activity of HBMSCs, FD and FE. *n* = 5, symbols refer to individual patients with three technical replicates. Results presented as mean +/- SD, statistics analyzed using a one-way ANOVA, significance presented as * < 0.05, ** < 0.01, *** < 0.001, **** < 0.0001.

HBMSCs from the femoral diaphysis and femoral epiphysis were analyzed for their ability to differentiate down the tri-lineage pathways. The cells were first assessed for both early and late markers of osteogenic differentiation. Cells were cultured in Osteogenic I media for seven days before the level of alkaline phosphatase (ALP), an early marker for osteogenic differentiation, was analyzed. Figure 2A shows representative images depicting ALP expression across the two HBMSC sources.

The Images show similar levels of ALP staining in the FD derived cells compared to those from the FE, with comparable results across multiple donor samples (*n* = 5, age range 13–90, mixed sex). The HBMSCs were also quantified for ALP expression, which showed that, while both FD and FE derived cells indicated an increase in ALP when cultured in osteogenic media compared to basal (Figure 2B), the osteogenic FD cells were elevated compared to the osteogenic FE cells, which was statistically significant in two of the patient samples. This correlation was also seen in the specific activity of ALP (Figure 2C), calculated by quantifying the level of DNA, which showed an increased expression in the FD derived cells compared to the FE cells that was significant in two patient samples. As cells from the FD region are known to have a higher percentage of hematopoietic cells than the FE [7,8], this could explain the differences found, although this was at an early time point and does not assess the effect on mature osteogenic differentiation and mineralization.

**Figure 3:**
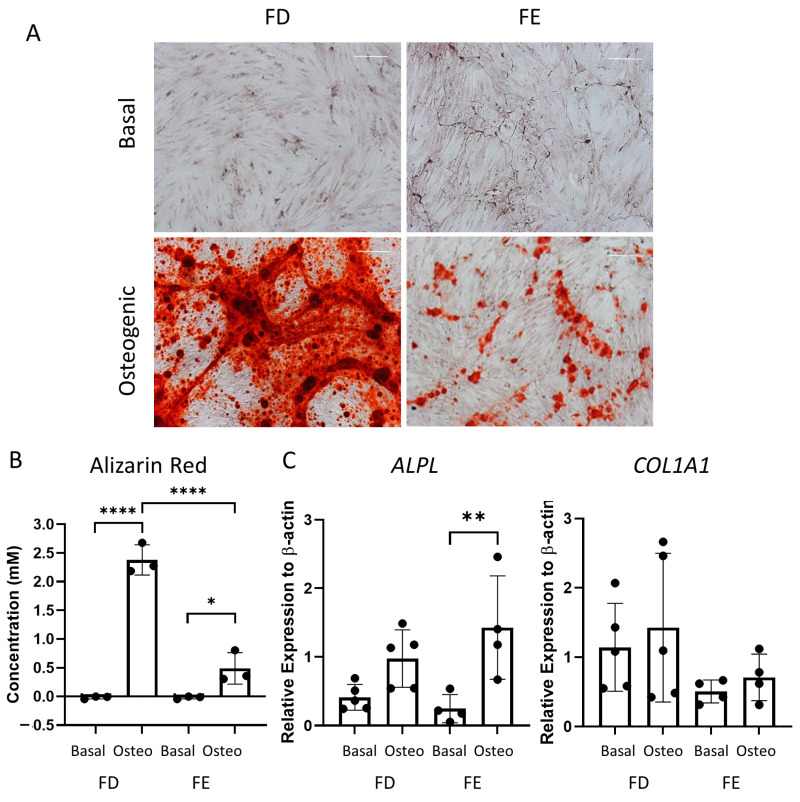
Osteogenic differentiation and mineralization of HBMSCs from two regions of the femur. FD and FE HBMSCs were cultured in basal or osteogenic II and mineralization media for 28 days. (**A**) Alizarin red stains areas of mineralization red, demonstrating osteogenic differentiation. Scale bar= 100 µm. (**B**) Alizarin red quantification was performed of the HBMSCs, absorbance analyzed at 405 nm. (**C**) Gene expression analysis of ALPL and COL1A1 as a relative expression to β-Actin for HBMSCs was performed by qPCR. *n* = 3–5, symbols refer to individual patient samples with three technical replicates. Results presented as mean +/− SD. Significance determined using a one-way ANOVA, significance presented as * < 0.05, ** <0.01, **** < 0.0001.

Following the differences seen in the early-stage osteogenic differentiation (Figure 2), the two sources of HBMSCs were also analyzed for their ability to mineralize after 28 days in osteogenic and mineralization media. The level of calcium deposit was detected using alizarin red staining (representative images, Figure 3A), which was then quantified. While the basal FD and FE derived HBMSCs demonstrated no alizarin red staining, the osteogenic FD cells showed a significantly higher concentration than the corresponding osteogenic FE cells in three donor samples (Figure 3B), indicating an increase in osteogenic differentiation. To confirm this, gene expression for ALP (*ALPL*) and collagen I (*COL1A1*) were determined by qPCR. *COL1A1* is an extracellular protein expressed during all stages of osteoblast differentiation [15]. When comparing gene expression on the HBMSCs, there was a high level of variability between the different donor samples (Figure 3C). There was a lower expression of *ALPL* in the FD derived cells compared with the FE. In contrast, there was an increased expression of *COL1A1* in the FD derived cells compared to the FE. This suggests that the cells from the FD region had differentiated more towards a mature osteogenic phenotype compared to the cells from the FE.

### 2.2. Adipogenic Differentiation of HBMSCs

**Figure 4:**
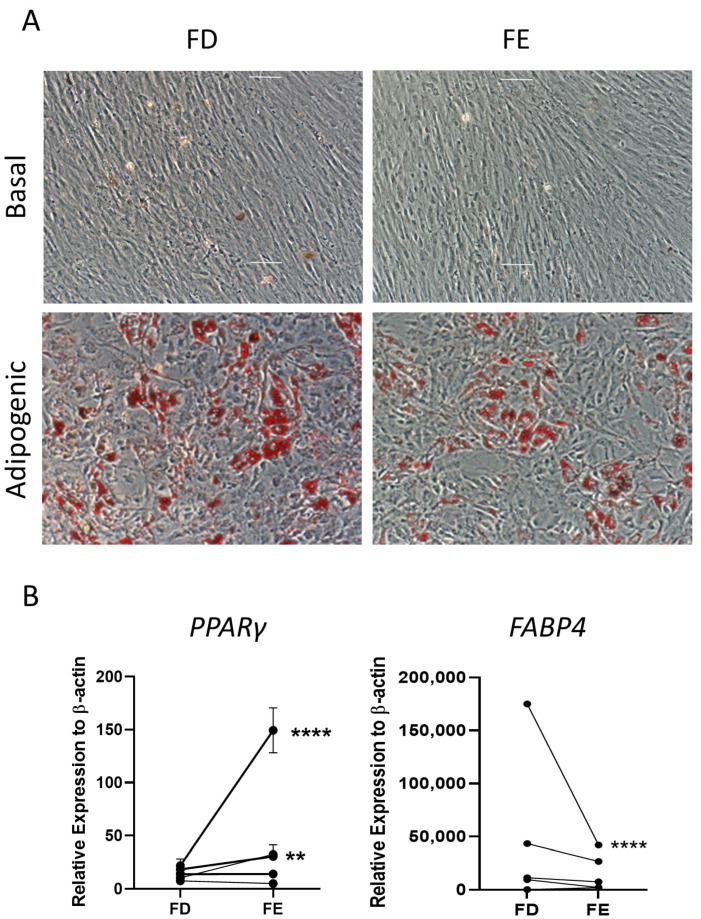
Adipogenic differentiation of HBMSCs from the two regions in the femur. FD and FE HBMSCs were cultured in basal or adipogenic media for 14 days. (**A**) Adipogenic cells produce lipid droplets which are stained by the oil red O (red). Scale bar = 100 µm. (**B**) Gene expression analysis of FABP4 and PPARγ in HBMSCs was performed by qPCR, *n* = 5, symbols refer to individual patient samples with three technical replicates. Results are presented as mean +/− SD, significance determined using a one-way ANOVA. For both statistical analysis significance presented as ** <0.01, **** < 0.0001.

The two sources of HBMSCs were then assessed for their ability to differentiate down the adipogenic lineage. These cells were stained with oil red O after 14-day culture, which stained lipid droplets secreted by the cells. As shown in Figure 4A, both FD and FE derived cells differentiated into adipogenic cells with comparable morphology and similar levels of staining. Gene expression for two markers of adipocyte differentiation, peroxisome proliferator-activated receptor γ (*PPARγ*), an early marker, and fatty acid binding protein 4 (*FABP4*), a late marker, were determined by qPCR for five HBMSC donors (Figure 4B). For three out of five donors, there was a higher level of *PPARγ* expression in the FE derived cells compared to the FD, where two of these donors showed a significantly higher expression. The remaining two donors demonstrated very similar, low levels of *PPARγ* in both FD and FE derived cells. In contrast, the cells from the FE showed a lower level of *FABP4* expression compared with cells from the FD, although this was only significant in one of the donor samples (F52). This suggests that the FD-derived HBMSCs had a more mature phenotype compared to the FE derived HBMSCs, reflecting a higher number of hematopoietic cells present in the FD region [7,8].

### 2.3. Chondrogenic Differentiation of HBMSCs

**Figure 5:**
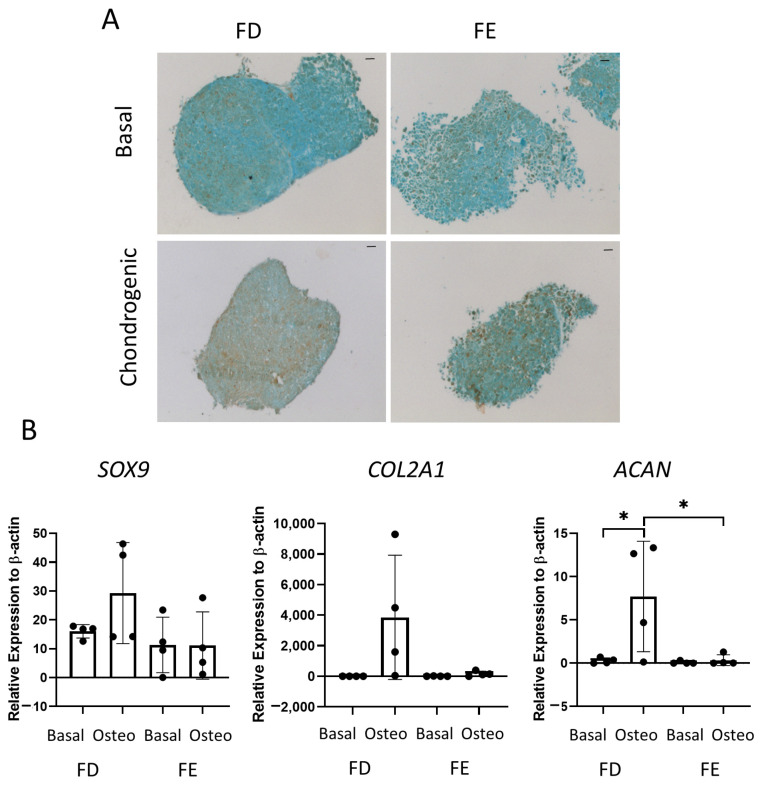
Chondrogenic differentiation of HBMSCs from two regions of the femur. Cell pellets of FD and FE HBMSCs were cultured in basal or chondrogenic media for 28 days. (**A**) SOX9 staining of basal and chondrogenic cultured cell pellets. SOX9 stained brown, proteoglycans stained blue with alcian blue, Scale bar = 100 µm. (**B**) Gene expression analysis of SOX9, aggrecan (ACAN), and collagen II (COL2A1) as a relative expression to β-Actin were performed by qPCR for FD and FE HBMSCs (*n* = 4, symbols refer to individual patient samples with three technical replicates). Results presented as +/− SD, significance determined using a one-way ANOVA, significance presented as * < 0.05.

The final tri-lineage differentiation pathway assessed was chondrogenic differentiation. Figure 5A shows representative images of SRY-Box Transcription Factor 9 (SOX9) staining in cell culture pellets, a transcription factor expressed in chondrocytes and linked to early cartilage development. Chondrocytes are routinely generated in spheroid culture, as monolayer culture has been shown to decrease chondrogenic potential [16]. An increase in SOX9 staining was seen in the FD derived chondrogenic pellet compared to the FE. Gene expression of three markers of chondrogenic differentiation, *COL2A1*, *SOX9*, and *ACAN*, were also determined by qPCR. *COL2A1* is one of the major components of the cartilage matrix [17], while *ACAN* is present in articular cartilage and is important in the structure and function of cartilage [18]. Figure 5B showed there was an increase in all three genes in the FD derived chondrogenic pellets compared with the FE, although this was only significant for *ACAN*. While a clear increase could be seen in all genes for FD derived chondrogenic pellets compared with basal control, there was large patient variability, and no difference was found in the FE-derived chondrogenic pellets compared to basal control. This suggested that, while the FD pellet cultures resulted in a mature chondrogenic phenotype, there was only very low levels of differentiation found in the FE samples.

### 2.4. Osteogenic Differentiation of Osteosarcoma Cell Lines

Clear tri-lineage differences were found between HBMSCs taken from the FD and FE regions of the femur. Osteosarcoma is known to develop near the growth plates of the bone in adolescents [19]. This is also where bone marrow conversion occurs, which may affect the initiation of the tumor itself. Osteosarcoma cell lines Saos-2 and MG63 were assessed for tri-lineage differentiation and compared to the HBMSC characteristics to determine whether there was a correlation between osteosarcoma lines and either ‘red’ (FD) or ‘yellow’ (FE) bone marrow. These two cell lines have been previously shown to have differing rates of tumor growth and metastasis development in vivo [20] and have also been characterized for tri-lineage differentiation [21], but they have not yet been compared to ‘red’ and ‘yellow’ HBMSCs.

**Figure 6:**
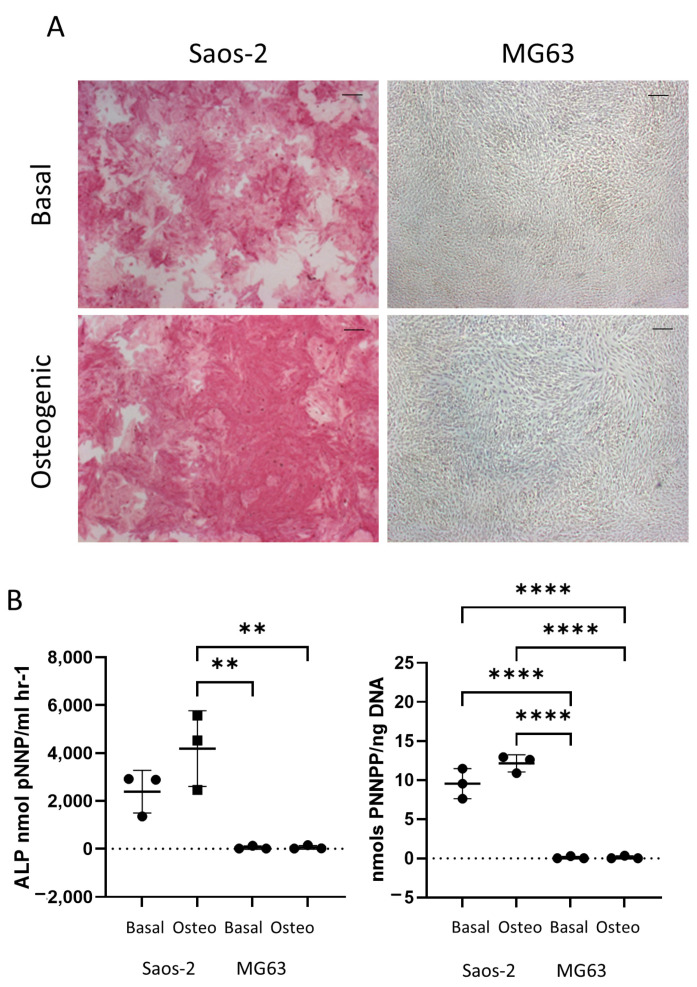
Early osteogenic differentiation of two osteosarcoma cell lines Saos-2 and MG63. (**A**) Representative images of ALP staining (pink/red) of two osteosarcoma cell lines Saos-2 and MG63. Cells were cultured in basal or osteogenic I media for seven days. Scale Bar = 100 µm. (**B**) ALP analysis and ALP specific activity osteosarcoma cell lines. *n* = 3, symbols refer to individual patient samples with three technical replicates. Results presented as mean +/− SD, statistics analyzed using a one-way ANOVA, significance presented as ** <0.01, **** < 0.0001.

Figure 6 shows the difference in early osteogenic potential of the osteosarcoma cell lines Saos-2 and MG63. These cells were cultured for seven days in basal or osteogenic I media, and then they were analyzed for ALP expression. The Saos-2 cell line had high levels of ALP staining in both basal and osteogenic I media (Figure 6A), while, in contrast, the MG63 cells had no ALP staining in either medium. This was supported by quantification of ALP signaling, where ALP and the specific activity (Figure 6B) showed high levels in the Saos-2 cells, but very low levels in the MG63 cells. The Saos-2 cells had an overall higher level of ALP expression compared to the HBMSCs, with the exception of one patient. This patient had a level of around 4000 ALP nmol pNNP/mL h^−1^ in their FD derived HBMSCs (Figure 2B), which was similar to the average expression level of the Saos-2 cells. The Saos-2 cells also had a similar specific activity to three of the FD derived patient samples (Figure 2C), ranging between 10–15 nmols pNNP/ng DNA. In contrast, the lack of ALP expression of the MG63 cells did not align with either of the HBMSC sources, suggesting this cell line could have potentially already matured down a different differentiation pathway.

**Figure 7:**
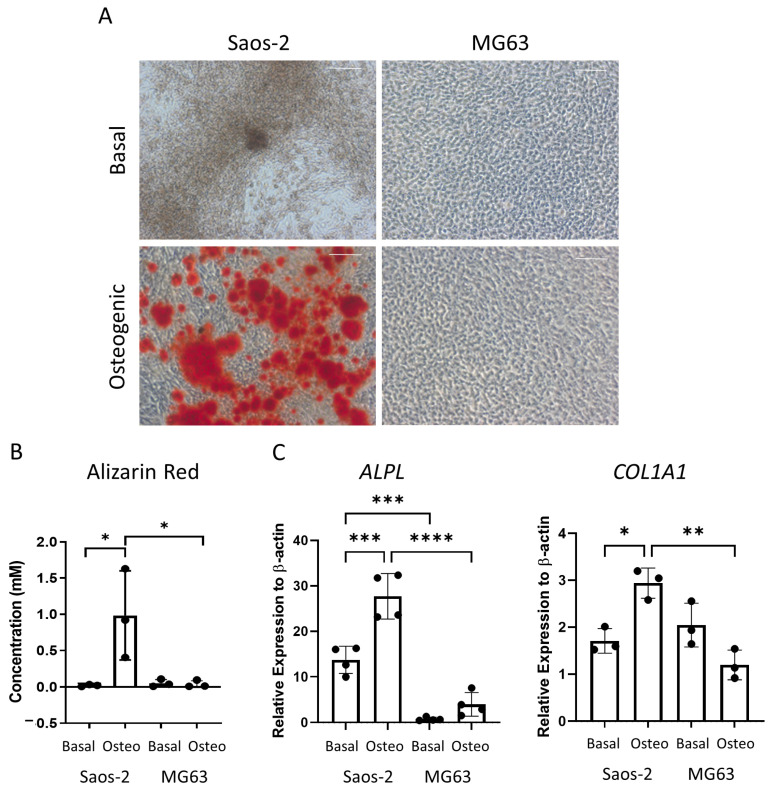
Osteogenic differentiation and mineralization of two osteosarcoma cell lines. Two osteosarcoma cell lines Saos-2 and MG63 were cultured in basal or osteogenic II and mineralization media for 28 days. (**A**) Alizarin red stains areas of mineralization red, demonstrating osteogenic differentiation. Scale bar = 100 µm. (**B**) Alizarin red quantification was performed on the osteosarcoma cell lines; absorbance was analyzed at 405 nm. (**C**) Gene expression analysis of ALPL and COL1A1 as a relative expression to β-Actin for osteosarcoma cell lines was performed by qPCR. *n* = 3–4, symbols refer to individual patient samples with three technical replicates. Results are presented as mean +/- SD. Significance determined using a one-way ANOVA, significance presented as * < 0.05, ** <0.01, *** < 0.001, **** < 0.0001.

The osteosarcoma cells were then assessed for late-stage osteogenic differentiation and mineralization. The cells were cultured in osteogenic II media for two weeks, followed by another two weeks incubation in mineralization media, and then they were either stained for alizarin red or lysed for qPCR analysis (Figure 7). The alizarin red staining demonstrated distinct differences, where the Saos-2 cells showed a high level of staining, while the MG63 cells were negative for alizarin red (Figure 7A). This pattern was also supported in the alizarin red quantification (Figure 7B), which was statistically higher in the Saos-2 cells compared to the MG63 cells. The concentration of the Saos-2 cells in Figure 7B (~1 mM) was found to be in between the concentrations determined by the FD (~2.2 mM) and FE (~0.5 mM) derived HBMSCs (Figure 3B). In contrast, the MG63 cells showed no staining, which did not align with either of the HBMSC sources.

The gene expression for *ALPL* and *COL1A1* (Figure 7C) was analyzed in the two osteosarcoma cell lines. Here, the Saos-2 cells had a higher level of both *ALPL* and *COL1A1* expression compared to the MG63 cells, although it was noted that the expression of *COL1A1* was higher in the basal incubated MG63 cells compared to the osteogenic incubated cells. The Saos-2 *ALPL* expression was also higher than both the FD and FE derived cells (Figure 3C), which showed a more similar expression in line with the MG63 cells. The Saos-2 cells also had a higher expression of *COL1A1* compared to the HBMSCs, which once again corresponded to the basal MG63 expression. This suggested that the Saos-2 cells showed increased levels of osteogenic differentiation compared to the two HBMSC sources. However, although there was no alizarin red staining of the MG63 cells, they had a similar gene expression profile to the two sources of HBMSCs.

### 2.5. Adipogenic Differentiation of Osteosarcoma Cells

**Figure 8:**
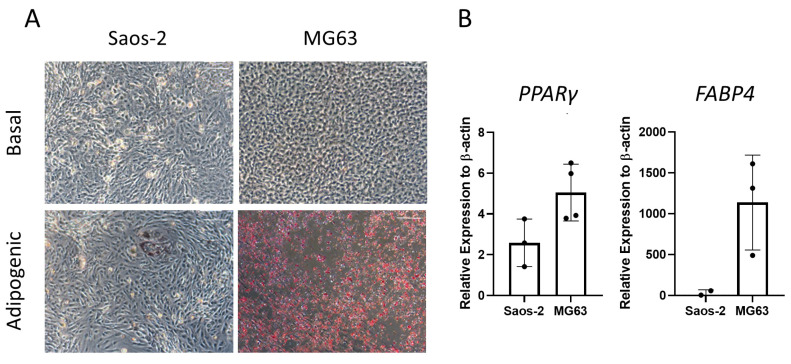
Adipogenic differentiation of two osteosarcoma cell lines. Two osteosarcoma cell lines Saos-2 and MG63 were cultured in basal or adipogenic media for 14 days. (**A**) Adipogenic cells produce lipid droplets, which are stained by the oil red O (red). Scale bar = 100 µm. (**B**) Gene expression analysis of FABP4 and PPARγ in the osteosarcoma cell lines was performed by qPCR, *n* = 3–4, symbols refer to individual patient samples with three technical replicates. Results are presented as mean +/− SD, significance determined using a one-way ANOVA, no significance found.

Next, the two osteosarcoma cell lines, Saos-2 and MG63, were assessed for adipogenic differentiation by culturing in adipogenic media for 14 days and staining with oil red O (Figure 8). The majority of Saos-2 cells were negative for oil red O staining (Figure 8A). However, in rare areas of positive staining, the morphology of the individual cells was comparable to the HBMSC, showing an increase in cell size. In contrast, the MG63 cells had a high level of staining, which was equally distributed across the well. These patterns of staining for both cell lines did not align with those found by the two HBMSC sources (Figure 4A), which both had higher levels compared to Saos-2 but lower than MG63. The cells were also analyzed for their gene expression of *PPARγ* and *FABP4*. The MG63 cells had a higher expression of both of these genes compared to the Saos-2 cells, which was also lower than the HBMSC gene expression (Figure 4B). This suggests that neither of the osteosarcoma cell lines aligned with the expression profile of the two sources of HBMSCs when assessed for adipogenic differentiation.

### 2.6. Chondrogenic Differentiation of Osteosarcoma Cells

**Figure 9:**
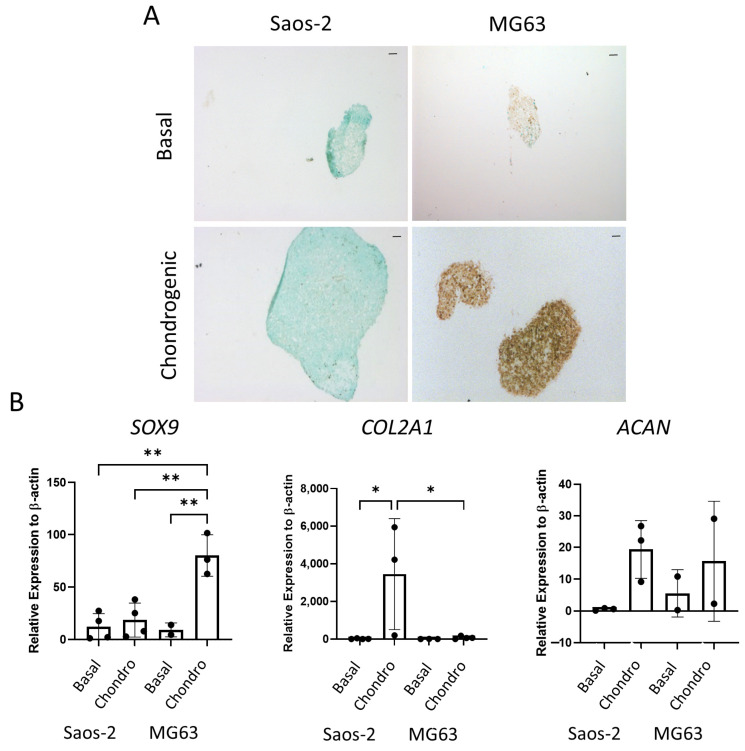
Chondrogenic differentiation of two osteosarcoma cell lines. Cell pellets of two osteosarcoma cell lines Saos-2 and MG63 were cultured in basal or chondrogenic media for 28 days. (**A**) SOX9 staining of basal and chondrogenic cultured cell pellets. SOX9 stained brown, proteoglycans stained blue with alcian blue, Scale bar = 100 µm. (**B**) Gene expression analysis of SOX9, ACAN and COL2A1 as a relative expression to β-Actin were performed by qPCR for the osteosarcoma cell lines (*n* = 2–3, symbols refer to individual patient samples with three technical replicates). Results are presented as +/− SD, significance was determined using a one-way ANOVA, significance was presented as * < 0.05, ** < 0.01.

The final tri-lineage pathway assessed for the osteosarcoma cell lines was the chondrogenic pathway. Cells were cultured as cell pellets in chondrogenic media for 21 days, then either embedded, sectioned, and stained for SOX9 or lysed for qPCR analysis. In Figure 9A, the Saos-2 osteosarcoma cells were negative for SOX9 staining, while the MG63 cells demonstrated an increased level of staining in the chondrogenic cultured pellets compared to the basal control. Similarly, gene expression of *SOX9* (Figure 9B) was low in the Saos-2 cells, but it was significantly higher in the MG63 cell line. In contrast, the *COL2A1* expression was significantly increased in the Saos-2 chondrogenic pellets compared with both the Saos-2 basal control and the MG63 chondrogenic pellets. *ACAN* expression was similar in both Saos-2 and MG63 chondrogenic pellets, and, for both cell lines, there was an increased trend in the chondrogenic pellet compared with the basal control. This suggests that both cell lines can differentiate down the chondrogenic pathway, but at different rates, with Saos-2 cells showing higher expression of the later stage marker *COL2A1*, while MG63 cells had a higher expression of the early marker *SOX9*. Compared to the chondrogenic differentiation of the two HBMSC sources in Figure 5, the Saos-2 cells had a lower *SOX9* expression, but a similar *COL2A1* expression to the FD derived cells, and a higher expression of *ACAN*. In contrast, the MG63 cells had a higher expression of *SOX9*, but a similarly low expression of *COL2A1* aligned with the FE derived cells, and conversely a higher expression of *ACAN.*

The tri-lineage differentiation expression of ‘red’ (FD) and ‘yellow’ (FE) bone marrow showed distinct differences. Disparities were also seen between the two osteosarcoma cell lines, Saos-2 and MG63. While the Saos-2 cells aligned with the FD derived cells in terms of osteogenic and chondrogenic potential, overall, the MG63 cell characterization did not correlate with the FD or FE derived HBMSCs expression.

## 3. Discussion

Osteosarcoma is most prevalent in children and adolescents, and it typically forms around the growth plate in long bones [19]. Ongoing research has shown that the cellular interactions within the tumor microenvironment of osteosarcoma, as in other cancers, are important in the development and growth of this disease, with manipulation of the microenvironment potentially offering novel therapeutic targets [22]. Conversion from ‘red’ to ‘yellow’ bone marrow gradually occurs during aging, reaching the metaphysis and growth plate during adolescence, which may play a key role in the initiation of osteosarcoma. Within the HBMSC population are human mesenchymal stem cells, and, although they are still not fully defined, they are characterized by the potential to differentiate into three cell lineages: osteoblasts, adipocytes, and chondrocytes [23,24]. This stem cell population has been previously compared in HBMSCs and adipose tissue [23], indicating differences in osteogenic and adipogenic differentiation, but it has not yet been fully compared in HBMSCs from differing skeletal locations, notably between ‘red’ and ‘yellow’ bone marrow. Similarities in tri-lineage differentiation between the HBMSCs and osteosarcoma cell lines may support a role in tumor development. In this report, we have shown that skeletal location in the femur affects the ability of the HBMSCs to differentiate down these tri-lineage pathways, with similarities in osteogenic and chondrogenic phenotype occurring between the ‘red’ bone marrow and the Saos-2 osteosarcoma cell line.

FD-derived cells were isolated from the diaphysis/metaphysis region of the femur, which has been described as consisting of a higher level of ‘red’ bone marrow and consequently has more hematopoietic cells than the FE derived. The HBMSCs from the FE were isolated from the epiphysis of the femur and have been described as consisting of a larger proportion of ‘yellow’ bone marrow and adipocytes [7,8]. In these studies, it was found that the FD-derived cells showed a higher potential for osteogenic differentiation in both the early and later stages compared to their corresponding FE derived cells (Figure 2 and Figure 3), although donor variability was evident. In the adipogenic analysis, oil red O staining of these cells suggested there were similar levels of adipogenic differentiation between the FD and FE derived cells (Figure 4). In contrast, gene expression of three out of five donors showed FE cells had an increased level of *PPARγ* compared with FD cells, with a corresponding decreased level of *FABP4*, suggesting the FD derived cells had progressed further down the adipocyte differentiation pathway, containing more mature adipocytes, while FE derived cells were still in the earlier stages of adipogenic differentiation. Chondrogenic differentiation of these cells also demonstrated a higher chondrogenic potential in the FD-derived cells compared to the corresponding HBMSCs from the FE region (Figure 5).

Osteogenic and chondrogenic differentiation are important for the continued repair and growth of the bone [25], and this can explain the increased expression found in cells from the hematopoietic region (FD), which is important in bone metabolism and the production of red and white blood cells, compared to cells from the adipocyte region (FE). It is important to note that these human bone marrow samples were received from patients needing hip replacements, which means they likely suffered from osteoporosis or osteoarthritis. This may have affected their tri-lineage ability, although the biological impact was minimized by using matching patient samples between the FD and FE regions.

Osteosarcoma routinely occurs in the metaphysis region of long bones near the growth plate [19]. This is the region that coverts from ‘red’ to ‘yellow’ bone marrow during puberty, and it is possible that mutation occurring during this process is involved with the development of osteosarcoma. The two osteosarcoma cell lines Saos-2 and MG63 have distinctly different tri-lineage differentiation abilities, with data showing the cell line Saos-2 had a high level of osteogenic differentiation, both at early and late time points (Figure 6 and Figure 7), low level of adipogenic differentiation (Figure 8), and a more mature chondrogenic differentiation expression (Figure 9). In contrast, the osteosarcoma cell line MG63 showed no osteogenic differentiation (Figure 6 and Figure 7), an increased level of adipogenic differentiation (Figure 8), and an earlier stage chondrogenic differentiation compared to the Saos-2 cells (Figure 9). High levels of serum ALP have been previously linked to a poorer prognosis in osteosarcoma patients [26,27], suggesting that the Saos-2 cell line was more aggressive than the MG63 cell line [20]. Compared with the MG63 cell line, Saos-2 cells showed a more similar expression profile with the two sources of HBMSCs, in particular with the FD derived cells, which had similar characteristics in osteogenic differentiation including ALP specific activity and *COL1A1* expression. The largest differences seen between the osteosarcoma cell lines and the two sources of HBMSCs was during adipogenic differentiation, with very different oil red O staining patterns (Figure 4 and Figure 8), as well as lower expression of adipogenic genes. MG63 cells specifically showed a very high level of oil red O staining, which did correlate with an increase in *FABP4* and *PPARγ*, but the level of expression for both of these genes are lower than for the HBMSCs. This could be due to high levels of Collagen III, which has been shown to be deposited by MG63 cells [28], which has also been linked with adipogenesis [29].

As osteosarcoma is believed to mutate from cells along the osteoblast differentiation pathway, this could explain the closer similarities between the Saos-2 cell line with the FD derived HBMSCs that underwent osteogenic differentiation, opposed to the FE-derived HBMSCs. Unfortunately, this does not explain the large tri-lineage differences between the Saos-2 and MG63 cell lines. These differences could be a result of using immortalized cell lines, which do not always resemble the primary tumor from which they were taken [30]. Future work characterizing the human bone marrow composition from the primary osteosarcoma samples could be a potential step to overcome these limitations. While this direction could further support the connection between bone marrow conversion and osteosarcoma initiation, these studies show clear differences between the tri-lineage differentiations of two sources of HBMSCs, which correlate with certain characteristics of two osteosarcoma cell lines.

## 4. Materials and Methods

### 4.1. Cell Culture

HBMSCs were removed from bone marrow taken from the femoral diaphysis (FD) and femoral epiphysis (FE), and they were isolated into a single cell suspension through vigorous washes in alpha-MEM media. Two osteosarcoma cell lines were used in this study (Saos-2 and MG63, both ATCC). All cell lines and primary cells were cultured in basal media (Table 1), at 37 °C in a humidified 5% CO_2_ incubator.

### 4.2. Osteogenic Differentiation

Cells were seeded in 12 well plates at 1–3 × 10^4^ cells per ml and cultured in basal or osteogenic I media (Table 1) for seven days. Cells were then fixed and stained for ALP, as previously described [31], or lysed in CelLytic M and stored at −20 °C for further ALP biochemical and specific activity analyses, as previously described [32].

To assess mineralization cells were seeded as above in osteogenic II media for 14 days then mineralization media for a further 14 days (Table 1). Cells were then lysed for qPCR analysis or stained for alizarin red, as previously described [33], and levels were quantified by analyzing the absorbance at 405 nm [34].

### 4.3. Adipogenic Differentiation

To assess adipogenic differentiation, cells were seeded as described above and cultured in basal or adipogenic media for 14 days (Table 1). Cells were then either lysed for qPCR analysis or stained with oil red O, as previously described [32].

### 4.4. Chondrogenic Differentiation

For chondrogenic assays, all cells were re-suspended at a concentration of 5 × 10^5^ cells per ml in either basal or chondrogenic media (Table 1) and centrifuged in 1.5 mL centrifuge tubes at 400 g for 10 min. Cell pellets were then cultured at 37 °C in a humidified 5% CO_2_ incubator with 5% O_2_ for 21 days, before being either lysed for qPCR analysis or fixed for staining. For histological staining, pellets were fixed in 4% PFA and dehydrated through increasing concentrations of ethanol and histoclear before embedding in paraffin. The chondrogenic cell pellets were sectioned (7 µm) then deparaffinized in histoclear and rehydrated through graded alcohols. Heat-induced antigen retrieval in citrate buffer (pH6) was performed at 90 °C for 20 min. Sections were then stained for SOX9, using the ImmPRESS Horse Anti-rabbit I IgG Plus polymer kit, following the manufacturer’s instructions.

### 4.5. qPCR Analysis

RNA extraction was performed on pre-lysed samples stored at −80 °C, using the ReliaPrep RNA Cell Miniprep System (Promega, Madison, WI, USA), according to the manufacturer’s protocol. The resulting RNA was measured using a Nanodrop 100 V3.8.1 (ThermoFisher Scientific, Waltham, MA, USA), and then it was stored at −80 °C. RNA was then reverse transcribed using a TaqMan Kit (Applied Biosystems, Waltham, MA, USA) with either 20 µL or 40 µL total reaction volume. The cDNA was then generated using a Verriti 96-well Thermal Cycler (ThermoFisher Scientific) using the following protocol: 10 min at 25 °C, 30 min at 48 °C then 5 min at 95 °C. The cDNA samples were stored at −20 °C, then analyzed by qPCR, with a GoTaq qPCR Master Mix (Promega). Master mix: 10 µL Power Sybr Green MM, 0.75 µL of 5 µM forward primer, 0.75 µL of 5 µM reverse Primer (Sigma, 100 µM stock), and 7.5 µL nuclease free water per well. 18 µL of the master mix was combined with 2 µL of cDNA, and qPCR was performed in a 7500 Real-Time PCR system (Applied Biosystems, Waltham, MA, USA), then it was analyzed using Applied Biosystems 7500 System SDS Software v2.0.5. A list of primers is provided in Table 2.

### 4.6. Statistical Analysis

Results were represented as means ± standard deviations. Significance was assessed using one-way analysis of variance (ANOVA) with Tukey’s post hoc test. Values of *p* ≤ 0.05 were considered significant. Significance was presented as * < 0.05, ** <0.01, *** < 0.001, **** < 0.0001.

## Figures and Tables

**Table 1:** Media Supplements.

Name	Media	Supplements
Basal	αMEM (Lonza)	10% FCS (Sigma, St. Louis, MO, USA), 1% P/S (100 U/mL Penicillin +100 µg/mL Streptomycin, Life technologies)
Osteogenic I	αMEM	10% FCS + 1% P/S +100 µM ascorbate acid 2-phosphate (Sigma) + 10 nM dexamethasone (Sigma)
Osteogenic II	αMEM	10% FCS +1% P/S + 50 µM Ascorbic acid 2-phosphate + 10 nM Vitamin D3 (Sigma)
Mineralization	αMEM	10% FCS +1% P/S + 50 µM Ascorbic acid 2-phosphate + 10 nM Dexamethasone + 2 mM Beta-Glycerol phosphate (Sigma)
Adipogenic	αMEM	+10 % FCS +1% P/S +100 mM Dexamethasone +0.5 mM IBMX (Sigma) +3 µg/mL ITS solution (Sigma) + 1 µM Rosiglitazone (Sigma)
Chondrogenic	αMEM	+1% P/S +100 µL ascorbic acid 2-phosphate +10 ng/mL TGF-β3 (Peprotech) +10 µg/mL ITS solution +10 nM Dexamethasone

**Table 2:** Primers used in qPCR analysis.

Gene	Protein	Forward 5′-3′	Reverse 5′3′
*ACTB*	βActin	GGCATCCTCACCCTGAAGTA	AGGTGTGGTGCCAGATTTTC
*PPARγ*	PPARγ	GGGCGATCTTGACAGGAAAG	GGGGGGTGATGTGTTTGAACTTG
*FABP4*	FABP4	TAGATGGGGGTGTCCTGGTA	CGCATTCCACCACCAGTT
*ALPL*	ALP	GGAACTCCTGACCCTTGACC	TCCTGTTCAGCTCGTACTGC
*COL1A1*	Collagen type I α1	GAGTGCTGTCCCGTCTGC	TTTCTTGGTCGGTGGGTG
*SOX9*	SOX9	CCCTTCAACCTCCCACACTA	TGGTGGTCGGTGTAGTCGTA
*COL2A1*	Collagen, type II α1	CCTGGTCCCCCTGGTCTTGG	CATCAAATCCTCCAGCCATC
*ACAN*	Aggrecan	GACGGCTTCCACCAGTGT	GTCTCCATAGCAGCCTTCC

## Data Availability

The data presented in this study are available in this research article.
